# Toxigenic *Corynebacterium diphtheriae* Infection in Cat, Texas, USA

**DOI:** 10.3201/eid2808.220018

**Published:** 2022-08

**Authors:** Ronald Tyler, Layda Rincon, Michael R. Weigand, Lingzi Xiaoli, Anna M. Acosta, Daniel Kurien, Hong Ju, Sonia Lingsweiler, Emilie Yvonne Prot

**Affiliations:** Texas Department of State Health Services, Harlingen, Texas, USA (R. Tyler Jr., E.Y. Prot);; Cameron County Public Health Department, San Benito, Texas, USA (L. Rincon);; Centers for Disease Control and Prevention, Atlanta, Georgia, USA (M.R. Weigand, A.M. Acosta, H. Ju);; IHRC, Inc., Atlanta (L. Xiaoli); ASRT, Inc., Atlanta (D. Kurien);; Texas A&M College of Veterinary Medicine, College Station, Texas, USA (S. Lingsweiler)

**Keywords:** toxigenic diphtheria, *Corynebacterium diphtheriae*, *C. diphtheriae*, domestic cat, cat diphtheria, Texas, United States

## Abstract

We report a toxigenic strain of *Corynebacterium diphtheriae* isolated from an oozing dermal wound in a pet cat in Texas, USA. We also describe the epidemiologic public health efforts conducted to identify potential sources of infection and mitigate its spread and the molecular and genetic studies performed to identify the bacterium.

Diphtheria, caused by toxigenic strains of the bacterium *Corynebacterium diphtheriae*, can result in life-threatening respiratory disease or cutaneous infections. Toxigenicity is contingent on successful bacterial expression of diphtheria toxin, encoded by a toxin gene (tox). Toxigenic *C. diphtheriae* is considered nearly exclusively a human pathogen, and humans are believed to be the reservoir. Because of high population coverage with diphtheria toxoid–containing vaccines, few diphtheria cases are reported in the United States. The most recently reported toxigenic infections were cutaneous and associated with international travel (*1*–*4*). 

A 2016 article reviewing available literature on *C. diphtheriae* isolated from animals identified 12 cases globally, 4 in dogs, 4 in cats, 2 in horses, 1 in a cow, and 1 in a fox. These infections were toxigenic only in 2 dogs and the 2 horses; 1 of the horses was identified in the United States (*5*,*6*). In contrast, toxigenic *Corynebacterium ulcerans* is a zoonotic organism that causes diphtheria-like illness in humans clinically indistinguishable from illness caused by toxigenic *C. diphtheriae*; it is more common than the diphtheria pathogen among household pets and their owners (*7*). 

To date, toxigenic diphtheria has not been detected in cats; however, nontoxigenic strains have been identified, including 2 from the ears of cats in the United States and 1 from the nose of a cat in Belgium (*8*,*9*). Although these 3 strains contained the tox gene, they were not toxin producing. Of note, the strains identified in the United States have recently been reclassified as a novel species, *C. rouxii*, because of biochemical and genetic differences with *C. diphtheriae* (*10*). 

Recommended public health response to toxigenic diphtheria infections in humans in the United States involves isolating and treating the index case-patient, identifying contacts, and vaccinating the patient and contacts with diphtheria toxoid–containing vaccine if it has been >5 years since the last dose (*11*). After treatment is completed, the index case-patient should be tested to confirm eradication of toxigenic *C. diphtheriae* and contacts monitored for development of diphtheria illness for 7–10 days after their most recent exposure; nasal and throat swab specimens should be collected to test for carriage, and prophylactic antibiotics should be administered. No formal recommendations exist for toxigenic diphtheria in animals because of its rarity, but health departments may pursue interventions similar to those to prevent transmission in humans. 

In October 2020, a veterinary clinic in southern Texas, USA, evaluated a male domestic shorthair cat 10 years of age for an oozing wound with multiple abscess pockets in its left flank. The clinic reported culturing *Mycobacterial farcinogenes* from a similar lesion on the cat in May 2018. A swab of the new wound was submitted for culturing to the Texas A&M Veterinary Medical Diagnostic Laboratory (College Station, TX, USA). The cat was empirically started on marbofloxacin, but after the owner reported worsening of the wound 2 days later, the attending veterinarian added amoxicillin/clavulanic acid to the regimen. The laboratory isolated 2 bacteria, *C. diphtheriae* and *M. farcinogenes*, and sent the *C. diphtheriae* isolate to the Centers for Disease Control and Prevention (CDC; Atlanta, GA, USA) for confirmation and toxigenicity testing. An investigation was conducted to identify possible exposures to toxigenic *C. diphtheriae*, identify potential human and animal carriers, and provide prevention measures. We report details of the investigation and subsequent molecular and genetic studies. 

## The Study

The owner of the index cat lived in a house with her husband and reported having no regular visitors the month before the cat developed the flank abscess. The owner reported they had 5 indoor-only cats, including the index cat, and 4 dogs that spent time both indoors and outdoors. The cat’s owner had received tetanus toxoid, reduced diphtheria toxoid, and acellular pertussis (Tdap) vaccine 3 years earlier; her husband provided no vaccine history. Cameron County Public Health (San Benito, TX, USA) collected an oropharyngeal swab specimen from the owner in November 2020 and submitted it to the Texas Department of State Health Services laboratory (Harlingen, TX, USA) for testing; the sample was negative for *C. diphtheriae*. Her husband did not submit a specimen. Both refused antibiotic prophylaxis, and the husband refused diphtheria toxoid–containing vaccine. 

The owners allowed oropharyngeal swab specimens to be collected from the 4 contact cats but refused to have their dogs tested. The cat samples were submitted to CDC but were negative for *C. diphtheriae* by isolation or PCR detection. One posttreatment swab specimen collected from the wound of the index cat in November was negative for *C. diphtheriae*. In December, the owner reported the wound appeared to be healing. 

Ten veterinary staff were identified as having potential exposures to the *C. diphtheriae* wound; 9 worked with the abscess wearing gloves and masks but no eye protection, and 1 was bitten while handling the cat. The Cameron County Public Health clinic collected oropharyngeal swab specimens from 9/10 exposed staff, and all tested negative for *C. diphtheriae* at the Texas Department of State Health Services laboratory. Six of 10 exposed staff received prophylactic antibiotics; 5/10 reported receiving no diphtheria toxoid vaccine within 5 years and so received vaccine boosters, and the remaining 5 reported having received diphtheria toxoid vaccine within 5 years. Human and animal contacts were assessed for clinical signs and symptoms, including skin lesions, consistent with diphtheria, but no signs or symptoms were observed. 

CDC conducted microbiologic and molecular characterization of the *C. diphtheriae* isolate (named PC1297), as described elsewhere (*12*,*13*). The isolate was confirmed as *C. diphtheriae* biotype gravis, and PCR confirmed presence of the tox gene. Modified Elek testing showed the isolate produced diphtheria toxin (*14*). The isolate was further characterized by whole-genome shotgun sequencing on an Illumina Miseq (https://www.illumina.com) (Appendix). Genome sequence-based multilocus sequence typing identified the isolate as ST705, unique among the 754 publicly available *C. diphtheriae* isolate sequences, and genome assembly confirmed presence of tox-encoding corynephage (Appendix) (*15*). Phylogenetic reconstruction of 273 *C. diphtheriae* isolate sequences, representing 270 unique sequence types and including 8 isolates from domestic animals. The results indicated that PC1297 was not related to isolates from previous cases reported in cats, including those now classified as *C. rouxii* (Appendix), nor was it closely related to any available human sequences; the nearest neighboring sequence in the phylogenic tree, ERR3932636, sequence type 669, was 6,948 single-nucleotide polymorphisms distant. 

## Conclusion

We report public health response to a rare case of cutaneous toxigenic diphtheria in a pet cat. Not all animal and human contacts could be tested, but *C. diphtheriae* was not detected among those tested; no source for the infection was identified. Comparative genomic analyses suggested that the identified strain differed from publicly available sequences of *C. diphtheriae*, including those from domestic pets, and the strain was not related to the neighboring *C. rouxii* sp. nov. Because of the limited availability of *C. diphtheriae* sequences from animals, there was insufficient data to determine whether the source of infection was from human or animal contact. Whereas our findings do not confirm whether animals might serve as reservoirs for diphtheria, they highlight the need for further study regarding transmission and environmental health. This case also reiterates the criticality of promptly discovering and identifying *C. diphtheriae* infections in companion animals for preventing spread of the disease to susceptible animals and humans. 

Appendix Additional information about toxigenic *Corynebacterium diphtheriae* infection in a cat.
