# Supplementary material for: Toxigenic Corynebacterium diphtheriae Infection in Cat, Texas, USA
Source: Emerg Infect Dis. 2022 Aug;28(8):1686–8. doi: 10.3201/eid2808.220018 (PMC9328917; doi:10.3201/eid2808.220018)
Supplement: Appendix — Additional information about toxigenic Corynebacterium diphtheriae infection in a cat. [file 22-0018-Techapp-s1.pdf]

Article DOI: https://doi.org/10.3201/eid2808.220018

Toxigenic *Corynebacterium diphtheriae* Infection in a Cat, Texas, USA

Appendix

Methods

C. diphtheriae PC1297 was grown on trypticase soy agar with 5% sheep blood at 37 °C for 24 h. Genomic DNA was extracted using the Maxwell RSC whole Blood DNA kit (Promega, https://www.promega.com) and the concentration was determined using the Qubit dsDNA broad range quantification kit (Thermo Fisher Scientific, https://www.thermofisher.com). Paired-end libraries (2 X 250bp) were prepared with the NEBNext Ultra DNA Library Prep Kit (New England Biolabs, https://www.neb.com) and sequenced on a Miseq using reagent v2, 500-cycle kit (Illumina, https://www.illumina.com). Raw Illumina read quality was assessed using FastQC v0.11.5 (*1*) before trimming and filtering with BBDuk (*2*) and Cutadapt (*3*). Trimmed reads were de novo assembled using SPAdes v3.9 (*4*) and evaluated with QUAST v4.5 (*5*).

Genome sequence-based molecular typing by multi-locus sequence typing (MLST) was determined from the assembled contigs according to stringMLST (*6*), and further confirmed with trimmed sequencing reads using SRST2 (*7*). The presence of diphtheria toxin homologs was determined by tBLASTn query of the assembled genomes using select references: Corynephage beta A and B subunits (NCBI accession no. P00588), Corynephage omega beta Diphtheria toxin (NCBI accession no. P00587), and Corynephage beta Diphtheria toxin homolog (NCBI accession no. P00589).

Publicly available *C. diphtheriae* genomic data for 273 isolates representing 270 unique sequence types were retrieved from the NCBI Sequence Read Archive (SRA) for phylogenetic comparison (Appendix Table 1). Raw reads were trimmed and filtered as described above. SNPs were determined by mapping all trimmed reads to the reference genome of NCTC13129 (NC_002935) using Snippy v4.3.8 with default settings (*8*). The phylogeny was reconstructed

from the resulting core SNP alignment using maximum-likelihood with RaxML v8.2.9 (*9*) and the tree was visualized with iToL v6 (*10*) (Appendix Figure 1).

References

1. Andrews S. FastQC: a quality control tool for high throughput sequencing data. 2010. [Accessed July 2021]. https://www.bioinformatics.babraham.ac.uk/projects/fastqc

2. Bushnell B. BBMap. [Accessed July 2021]. http://sourceforge.net/projects/bbmap

3. Martin M. Cutadapt removes adapter sequences from high-throughput sequencing reads. EMBnet J. 2011;17:10–2. https://doi.org/10.14806/ej.17.1.200

4. Prjibelski A, Antipov D, Meleshko D, Lapidus A, Korobeynikov A. Using SPAdes de novo assembler. Curr Protoc Bioinformatics. 2020;70:e102. PubMed https://doi.org/10.1002/cpbi.102

5. Gurevich A, Saveliev V, Vyahhi N, Tesler G. QUAST: quality assessment tool for genome assemblies. Bioinformatics. 2013;29:1072–5. PubMed https://doi.org/10.1093/bioinformatics/btt086

6. Gupta A, Jordan IK, Rishishwar L. stringMLST: a fast k-mer based tool for multilocus sequence typing. Bioinformatics. 2017;33:119–21. PubMed https://doi.org/10.1093/bioinformatics/btw586

7. Inouye M, Dashnow H, Raven LA, Schultz MB, Pope BJ, Tomita T, et al. SRST2: rapid genomic surveillance for public health and hospital microbiology labs. Genome Med. 2014;6:90. PubMed https://doi.org/10.1186/s13073-014-0090-6

8. Seemann T. Rapid haploid variant calling and core genome alignment. [Accessed July 2021]. https://github.com/tseemann/snippy

9. Stamatakis A. RAxML version 8: a tool for phylogenetic analysis and post-analysis of large phylogenies. Bioinformatics. 2014;30:1312–3. PubMed https://doi.org/10.1093/bioinformatics/btu033

10. Letunic I, Bork P. Interactive Tree Of Life (iTOL) v4: recent updates and new developments. Nucleic Acids Res. 2019;47(W1):W256–9. PubMed https://doi.org/10.1093/nar/gkz239

Appendix Table. *C. diphtheriae* isolate sequences used for phylogenetic analysis*

ID	atpA	dnaE	dnaK	fusA	leuA	odhA	rpoB	ST	Species (animal)†
ERR2094322	2	1	4	1	3	3	5	97	*C. diphtheriae*
ERR2094324	2	26	55	29	3	3	2	184	*C. diphtheriae*
ERR2094326	3	2	4	30	3	3	2	251	*C. diphtheriae*
ERR2094331	28	1	20	19	3	16	20	102	*C. diphtheriae*
ERR2094333	30	23	63	25	3	3	28	217	*C. diphtheriae*
ERR2094334	4	11	13	7	3	56	27	432	*C. diphtheriae*
ERR2094336	28	2	60	30	3	3	3	367	*C. diphtheriae*
ERR2094337	2	1	20	19	42	16	31	412	*C. diphtheriae*
ERR2167138	2	1	20	16	3	3	5	83	*C. diphtheriae*
ERR2173761	4	1	102	4	5	5	35	503	*C. diphtheriae*
ERR2173765	2	51	9	60	8	3	2	486	*C. diphtheriae*
ERR2173766	30	1	93	25	3	3	6	398	*C. diphtheriae*
ERR2173767	4	46	4	1	5	3	2	473	*C. diphtheriae*
ERR2173768	22	4	60	62	3	1	4	505	*C. diphtheriae*
ERR2173771	4	10	3	1	7	3	13	469	*C. diphtheriae*
ERR2173772	3	53	32	19	3	4	4	506	*C. diphtheriae*
ERR2173773	2	2	60	2	3	3	2	474	*C. diphtheriae*
ERR2173774	30	10	102	4	3	3	35	502	*C. diphtheriae*
ERR2217012	2	3	3	2	7	3	3	406	*C. diphtheriae*
ERR2217013	2	2	9	25	3	3	2	302	*C. diphtheriae*
ERR2217014	1	1	88	1	29	52	4	379	*C. diphtheriae*
ERR2217016	4	21	29	1	3	3	3	120	*C. diphtheriae*
ERR2217017	13	4	8	44	3	3	13	433	*C. diphtheriae*
ERR3331350	16	4	8	1	2	30	9	151	*C. diphtheriae*
ERR3331352	3	2	4	19	3	16	9	455	*C. diphtheriae*
ERR3331354	5	2	7	1	3	5	8	10	*C. diphtheriae*
ERR3331355	3	2	3	6	3	3	2	67	*C. diphtheriae*
ERR3331356	16	8	50	1	2	30	9	161	*C. diphtheriae*
ERR3331365	3	3	4	2	3	3	3	209	*C. diphtheriae*
ERR3932476	2	1	33	28	11	2	2	82	*C. diphtheriae*
ERR3932480	2	1	28	4	39	4	9	222	*C. diphtheriae*
ERR3932481	2	1	32	19	13	3	6	80	*C. diphtheriae*
ERR3932482	2	10	17	13	40	2	4	223	*C. diphtheriae*
ERR3932483	35	10	33	19	13	3	6	192	*C. diphtheriae*
ERR3932485	2	4	3	1	7	37	4	225	*C. diphtheriae*
ERR3932487	13	29	66	44	1	1	6	227	*C. diphtheriae*
ERR3932488	2	1	2	1	7	3	9	233	*C. diphtheriae*
ERR3932491	22	18	6	1	3	1	4	116	*C. diphtheriae*
ERR3932492	5	6	7	6	6	3	8	25	*C. diphtheriae*
ERR3932495	3	1	16	4	13	3	5	146	*C. diphtheriae*
ERR3932496	2	9	4	1	30	28	2	131	*C. diphtheriae*
ERR3932497	2	3	3	19	13	3	6	132	*C. diphtheriae*
ERR3932498	3	2	5	1	27	5	9	150	*C. diphtheriae*
ERR3932499	33	15	4	1	3	3	2	140	*C. diphtheriae*
ERR3932500	1	2	9	7	8	3	2	183	*C. diphtheriae*
ERR3932502	2	1	45	1	5	3	9	123	*C. diphtheriae*
ERR3932504	3	2	4	3	4	4	4	4	*C. diphtheriae*
ERR3932505	29	19	25	1	28	6	2	118	*C. diphtheriae*
ERR3932506	2	1	33	28	11	2	3	147	*C. diphtheriae*
ERR3932507	19	9	4	2	3	3	2	121	*C. diphtheriae*
ERR3932508	3	4	3	1	4	3	2	11	*C. diphtheriae*
ERR3932510	2	10	9	1	3	27	2	130	*C. diphtheriae*
ERR3932511	19	8	11	9	3	3	5	665	*C. diphtheriae*
ERR3932512	2	4	4	1	3	3	7	9	*C. diphtheriae*
ERR3932515	16	4	8	1	7	16	9	46	*C. diphtheriae*
ERR3932516	7	8	12	10	10	6	4	666	*C. diphtheriae*
ERR3932517	32	22	45	5	18	3	6	133	*C. diphtheriae*
ERR3932518	30	4	28	25	3	3	6	135	*C. diphtheriae*
ERR3932519	3	2	1	1	13	3	2	152	*C. diphtheriae*
ERR3932520	4	11	4	19	3	29	9	134	*C. diphtheriae*
ERR3932521	36	10	3	1	7	3	22	165	*C. diphtheriae*
ERR3932523	8	2	16	1	3	3	12	26	*C. diphtheriae*
ERR3932524	3	4	3	6	3	3	3	141	*C. diphtheriae*
ERR3932525	8	3	19	2	7	3	3	40	*C. diphtheriae*
ERR3932526	4	1	49	5	11	3	6	148	*C. diphtheriae*
ERR3932527	4	2	2	1	2	2	2	155	*C. diphtheriae*
ERR3932528	2	10	24	1	3	31	2	157	*C. diphtheriae*
ERR3932529	4	20	44	33	29	1	21	119	*C. diphtheriae*

ID	atpA	dnaE	dnaK	fusA	leuA	odhA	rpoB	ST	Species (animal)†
ERR3932530	3	2	13	35	3	2	4	149	*C. diphtheriae*
ERR3932532	14	2	23	4	2	14	2	44	*C. diphtheriae*
ERR3932533	3	2	3	1	33	16	18	154	*C. diphtheriae*
ERR3932534	3	3	3	2	3	3	3	3	*C. diphtheriae*
ERR3932537	3	5	6	5	3	3	6	8	*C. diphtheriae*
ERR3932538	3	4	4	1	3	3	5	117	*C. diphtheriae*
ERR3932539	16	4	8	1	3	30	9	158	*C. diphtheriae*
ERR3932540	4	2	5	2	3	3	5	6	*C. diphtheriae*
ERR3932541	32	1	45	5	11	3	6	145	*C. diphtheriae*
ERR3932542	1	1	1	1	1	78	1	671	*C. diphtheriae*
ERR3932543	9	4	13	11	3	3	9	21	*C. diphtheriae*
ERR3932545	4	21	2	1	3	3	3	127	*C. diphtheriae*
ERR3932546	31	4	48	1	7	1	9	129	*C. diphtheriae*
ERR3932547	2	4	8	1	7	3	9	258	*C. diphtheriae*
ERR3932548	19	9	46	19	3	26	22	126	*C. diphtheriae*
ERR3932549	22	4	138	1	18	3	4	672	*C. diphtheriae*
ERR3932554	30	4	35	25	11	3	6	124	*C. diphtheriae*
ERR3932555	2	10	29	1	12	2	13	143	*C. diphtheriae*
ERR3932557	2	1	39	19	13	24	13	96	*C. diphtheriae*
ERR3932558	2	1	6	29	3	3	2	91	*C. diphtheriae*
ERR3932560	3	1	5	2	3	23	2	88	*C. diphtheriae*
ERR3932564	2	1	38	29	3	3	2	87	*C. diphtheriae*
ERR3932568	19	10	61	4	5	5	2	206	*C. diphtheriae*
ERR3932569	2	4	4	29	3	16	2	205	*C. diphtheriae*
ERR3932570	3	1	13	16	38	2	9	212	*C. diphtheriae*
ERR3932574	2	10	56	45	11	3	3	228	*C. diphtheriae*
ERR3932575	2	1	9	19	3	17	2	667	*C. diphtheriae*
ERR3932576	2	1	9	29	3	3	2	237	*C. diphtheriae*
ERR3932579	3	4	67	29	3	16	2	235	*C. diphtheriae*
ERR3932580	2	9	6	29	3	3	2	238	*C. diphtheriae*
ERR3932581	11	1	20	16	3	3	14	239	*C. diphtheriae*
ERR3932584	2	9	9	29	3	3	2	241	*C. diphtheriae*
ERR3932585	32	1	70	19	3	3	18	250	*C. diphtheriae*
ERR3932587	13	2	44	41	29	1	21	366	*C. diphtheriae*
ERR3932589	2	9	4	59	3	3	2	424	*C. diphtheriae*
ERR3932590	3	2	4	1	3	3	13	136	*C. diphtheriae*
ERR3932594	3	4	11	2	3	3	2	426	*C. diphtheriae*
ERR3932595	22	2	4	29	3	40	2	268	*C. diphtheriae*
ERR3932600	3	2	73	4	43	22	2	274	*C. diphtheriae*
ERR3932601	28	2	38	19	13	3	6	269	*C. diphtheriae*
ERR3932602	2	8	63	30	85	3	19	673	*C. diphtheriae*
ERR3932603	2	10	69	4	5	11	2	668	*C. diphtheriae*
ERR3932604	4	4	96	7	86	57	32	674	*C. diphtheriae*
ERR3932606	11	1	85	2	3	3	2	356	*C. diphtheriae*
ERR3932608	22	9	43	7	3	3	2	109	*C. diphtheriae*
ERR3932611	4	1	20	19	13	16	9	361	*C. diphtheriae*
ERR3932612	25	1	6	19	3	16	6	425	*C. diphtheriae*
ERR3932613	2	23	32	1	55	5	43	362	*C. diphtheriae*
ERR3932614	28	12	6	19	3	51	6	352	*C. diphtheriae*
ERR3932616	2	1	61	19	3	16	2	355	*C. diphtheriae*
ERR3932617	24	15	86	19	11	3	18	374	*C. diphtheriae*
ERR3932618	3	2	35	1	18	3	2	86	*C. diphtheriae*
ERR3932621	46	10	60	7	3	3	2	375	*C. diphtheriae*
ERR3932624	3	8	9	1	3	40	2	310	*C. diphtheriae*
ERR3932625	3	1	12	1	42	16	31	259	*C. diphtheriae*
ERR3932626	2	26	4	4	62	3	2	421	*C. diphtheriae*
ERR3932627	3	8	8	30	3	4	19	95	*C. diphtheriae*
ERR3932630	1	2	9	57	3	3	2	419	*C. diphtheriae*
ERR3932632	3	2	70	1	7	3	2	595	*C. diphtheriae*
ERR3932633	3	3	92	2	3	3	3	387	*C. diphtheriae*
ERR3932634	2	2	38	58	3	3	22	422	*C. diphtheriae*
ERR3932636	18	10	27	21	20	5	16	669	*C. diphtheriae*
ERR3932638	2	2	4	1	3	3	46	388	*C. diphtheriae*
ERR3932639	3	8	9	30	3	4	19	413	*C. diphtheriae*
ERR3932640	2	1	61	4	5	5	2	423	*C. diphtheriae*
ERR3932642	3	1	38	4	3	3	2	410	*C. diphtheriae*
ERR3932643	2	2	2	3	11	64	4	536	*C. diphtheriae*
ERR3932644	4	2	104	30	3	51	2	511	*C. diphtheriae*
ERR3932645	2	26	55	29	30	3	2	414	*C. diphtheriae*

ID	atpA	dnaE	dnaK	fusA	leuA	odhA	rpoB	ST	Species (animal)†
ERR3932647	22	2	4	16	3	40	2	415	*C. diphtheriae*
ERR3932648	25	16	35	44	3	3	28	416	*C. diphtheriae*
ERR3932649	2	2	42	31	11	3	4	100	*C. diphtheriae*
ERR3932651	2	8	32	19	11	17	18	429	*C. diphtheriae*
ERR3932652	19	1	95	2	3	16	6	420	*C. diphtheriae*
ERR3932653	2	8	4	1	3	3	6	430	*C. diphtheriae*
ERR3932655	4	1	2	19	25	16	18	101	*C. diphtheriae*
ERR3932656	4	12	4	1	18	3	13	308	*C. diphtheriae*
ERR3932657	3	9	4	1	3	3	5	417	*C. diphtheriae*
ERR3932658	2	1	60	4	3	3	18	431	*C. diphtheriae*
ERR3932660	30	2	32	19	24	16	9	418	*C. diphtheriae*
ERR3932661	2	1	20	19	3	3	14	295	*C. diphtheriae*
ERR3932662	2	2	92	13	8	3	5	411	*C. diphtheriae*
ERR3932667	19	32	38	82	44	2	20	675	*C. diphtheriae*
ERR3932668	1	2	9	7	73	3	2	549	*C. diphtheriae*
ERR3932673	1	48	139	7	3	57	9	676	*C. diphtheriae*
ERR3932674	13	16	35	44	29	3	28	517	*C. diphtheriae*
ERR3932676	2	1	6	1	3	40	14	481	*C. diphtheriae*
ERR3932678	2	2	3	2	3	3	5	519	*C. diphtheriae*
ERR3932680	3	5	5	2	3	23	20	520	*C. diphtheriae*
ERR3932681	2	1	111	1	5	40	2	532	*C. diphtheriae*
ERR3932682	48	44	88	1	60	1	6	380	*C. diphtheriae*
ERR3932685	33	57	112	44	3	1	13	533	*C. diphtheriae*
ERR3932686	1	1	32	1	70	5	30	522	*C. diphtheriae*
ERR3932687	4	55	5	19	5	59	2	521	*C. diphtheriae*
ERR3932690	2	1	55	25	55	3	2	490	*C. diphtheriae*
ERR3932693	13	29	108	64	60	1	23	524	*C. diphtheriae*
ERR3932697	2	1	47	19	13	3	6	128	*C. diphtheriae*
ERR3932700	2	4	101	7	3	3	32	487	*C. diphtheriae*
ERR3932701	28	9	4	64	3	3	2	526	*C. diphtheriae*
ERR3932702	4	2	109	65	3	51	2	527	*C. diphtheriae*
ERR3932705	5	2	70	19	33	3	53	530	*C. diphtheriae*
ERR3932707	3	1	9	5	3	4	36	320	*C. diphtheriae*
ERR3932708	3	2	73	4	4	22	4	427	*C. diphtheriae*
ERR4332857	3	1	20	7	13	54	2	724	*C. diphtheriae*
ERR4332868	3	1	20	2	13	54	2	384	*C. diphtheriae*
ERR4332886	4	46	4	1	3	3	2	447	*C. diphtheriae*
ERR4332890	2	9	96	2	3	3	4	725	*C. diphtheriae*
ERR4332892	2	12	4	1	3	3	2	292	*C. diphtheriae*
SRR10054224	2	9	1	1	13	3	2	76	*C. diphtheriae*
SRR10054230	3	1	150	19	13	3	48	739	*C. diphtheriae*
SRR11184121	3	1	107	19	13	3	48	652	*C. diphtheriae*
SRR11184124	2	4	6	19	13	3	6	625	*C. diphtheriae*
SRR11184125	2	1	76	19	13	24	13	317	*C. diphtheriae*
SRR11184130	2	1	20	19	42	16	13	244	*C. diphtheriae*
SRR11184131	2	4	137	81	13	3	6	662	*C. diphtheriae*
SRR11184133	3	46	3	6	30	3	2	439	*C. diphtheriae*
SRR11184136	38	1	77	4	5	3	9	319	*C. diphtheriae*
SRR11184138	33	2	4	1	3	3	2	655	*C. diphtheriae*
SRR11184142	3	1	45	1	5	3	9	654	*C. diphtheriae*
SRR11184144	4	1	136	5	11	1	6	661	*C. diphtheriae*
SRR11184153	3	2	131	71	7	2	4	653	*C. diphtheriae*
SRR11184159	2	1	35	29	3	3	2	623	*C. diphtheriae*
SRR11184162	2	1	45	46	5	16	9	246	*C. diphtheriae*
SRR11184166	2	3	92	2	3	3	3	658	*C. diphtheriae*
SRR11184167	3	1	18	4	13	3	5	32	*C. diphtheriae*
SRR11184172	2	3	38	64	7	3	2	657	*C. diphtheriae*
SRR11184173	2	1	35	19	13	24	13	257	*C. diphtheriae*
SRR11184176	1	9	46	19	3	3	22	656	*C. diphtheriae*
SRR12235651 (PC0112)	2	2	4	1	3	3	2	50	*C. diphtheriae*
SRR12235663	3	2	4	15	4	4	4	31	*C. diphtheriae*
SRR12235668 (PC0153)	2	10	24	1	3	3	2	53	*C. diphtheriae*
SRR12235672 (PC0132)	2	10	29	1	12	10	13	38	*C. diphtheriae*
SRR12235677 (PC0104)	10	8	16	14	10	3	9	30	*C. diphtheriae*

ID	atpA	dnaE	dnaK	fusA	leuA	odhA	rpoB	ST	Species (animal)†
SRR12270034 (PC0652)	3	2	60	83	3	3	2	NT	*C. diphtheriae*
SRR12270040 (PC0646)	3	2	60	30	3	3	2	445	*C. diphtheriae*
SRR6816560	59	4	8	53	3	5	13	0	*C. diphtheriae*
SRR6816561	1	16	32	1	87	1	4	687	*C. diphtheriae*
SRR6816562	49	4	8	53	3	5	13	729	*C. diphtheriae*
SRR6816563	32	1	45	45	11	3	3	240	*C. diphtheriae*
SRR6816564	3	4	8	1	18	3	9	498	*C. diphtheriae*
SRR6816565	2	4	4	1	3	3	5	5	*C. diphtheriae*
SRR6816566	1	1	88	44	97	89	13	749	*C. diphtheriae*
SRR6816567	13	29	108	64	60	1	65	744	*C. diphtheriae*
SRR6816568	2	1	33	28	11	2	22	516	*C. diphtheriae*
SRR6816570	4	1	8	1	11	3	6	122	*C. diphtheriae*
SRR6816572	18	1	27	21	20	5	16	59	*C. diphtheriae*
SRR6816573	1	44	9	1	26	69	4	605	*C. diphtheriae*
SRR6816574	13	2	46	19	60	26	13	730	*C. diphtheriae*
SRR6816576	19	1	9	1	18	88	13	745	*C. diphtheriae*
SRR6816578	2	1	35	4	37	3	9	731	*C. diphtheriae*
SRR6816579	25	1	33	28	11	3	22	737	*C. diphtheriae*
SRR6816582	9	44	88	1	60	1	6	734	*C. diphtheriae*
SRR6816583	1	2	66	88	93	5	21	738	*C. diphtheriae*
SRR6816585	2	2	13	1	3	3	13	727	*C. diphtheriae*
SRR6816587	13	2	44	1	95	1	13	747	*C. diphtheriae*
SRR6816590	13	2	32	33	93	1	21	748	*C. diphtheriae*
SRR6816591	49	4	9	53	3	5	13	381	*C. diphtheriae*
SRR6816592	25	16	35	46	2	3	4	732	*C. diphtheriae*
SRR6816596	13	1	20	1	3	5	2	733	*C. diphtheriae*
SRR6816598	3	1	3	1	3	5	13	735	*C. diphtheriae*
SRR6816599	3	1	152	4	13	3	5	750	*C. diphtheriae*
SRR6816601	8	3	3	2	7	3	3	20	*C. diphtheriae*
SRR6816606	1	1	33	1	13	3	6	736	*C. diphtheriae*
SRR7039157	2	12	54	13	3	3	4	297	*C. diphtheriae*
SRR7039160	3	8	94	4	3	4	2	494	*C. diphtheriae*
SRR7039165	19	11	4	19	3	29	9	495	*C. diphtheriae*
SRR7039167	4	2	4	1	3	3	2	446	*C. diphtheriae*
SRR7039176	4	2	4	19	3	16	2	496	*C. diphtheriae*
SRR7039185	3	2	4	1	3	3	2	389	*C. diphtheriae*
SRR7039187	3	1	4	19	13	3	48	441	*C. diphtheriae*
SRR7039194	2	1	20	19	69	3	14	509	*C. diphtheriae*
SRR7039204	2	1	32	19	13	3	50	508	*C. diphtheriae*
SRR7039222	3	1	12	1	68	3	31	507	*C. diphtheriae*
SRR7223801	19	4	8	1	3	3	13	125	*C. diphtheriae*
SRR7825350	22	54	104	16	3	2	2	512	*C. diphtheriae*
SRR7825351	4	4	105	1	3	3	2	513	*C. diphtheriae*
SRR7825371	8	8	3	1	7	3	2	497	*C. diphtheriae*
SRR7825427	3	1	94	1	3	3	2	500	*C. diphtheriae*
SRR8417825	2	12	125	1	3	3	2	586	*C. diphtheriae*
PC1297	18	10	30	21	20	5	16	705	*C. diphtheriae* (cat)
PC0406	3	1	110	19	20	61	16	528	*C. diphtheriae* (dog)
ERR3574992	37	25	53	39	36	35	17	181	*C. rouxii*
ERR3574995	37	25	113	66	72	35	17	538	*C. rouxii*
SRR12235660 (PC0231)	20	14	31	26	22	21	17	74	*C. rouxii* (cat)
SRR12235661 (PC0230)	20	14	31	26	22	21	17	74	*C. rouxii* (cat)
SRR12235662 (PC0229)	20	14	31	26	22	21	17	74	*C. rouxii* (cat)
SRR12235664 (PC0226)	20	14	31	26	22	21	17	74	*C. rouxii* (cat)
PC0751	37	25	91	54	61	21	17	537	*C. rouxii* (dog)
PC1315	37	25	91	26	83	35	3	0	*C. rouxii* (dog)
ERR2094320	6	17	10	12	9	7	11	107	*C. belfantii*
ERR2094321	6	7	10	8	34	13	10	177	*C. belfantii*
ERR2094323	6	7	10	12	9	12	10	106	*C. belfantii*
ERR2094328	6	7	10	18	56	12	11	365	*C. belfantii*
ERR2094329	6	7	10	8	9	13	10	92	*C. belfantii*
ERR2757918	6	7	62	37	9	12	10	208	*C. belfantii*
SRR11184122	6	7	134	63	9	7	10	659	*C. belfantii*

ID	atpA	dnaE	dnaK	fusA	leuA	odhA	rpoB	ST	Species (animal)†
SRR11184123	6	7	10	12	9	7	10	81	*C. belfantii*
SRR11184129	6	7	10	12	84	13	15	663	*C. belfantii*
SRR11184141	6	7	10	18	9	13	10	170	*C. belfantii*
SRR11184154	6	7	135	8	9	77	10	660	*C. belfantii*
SRR11184155	6	7	21	12	9	12	11	409	*C. belfantii*
SRR11184157	6	7	10	37	9	12	11	353	*C. belfantii*
SRR11184171	6	7	10	37	9	12	10	226	*C. belfantii*
SRR7039177	6	7	21	61	9	7	10	501	*C. belfantii*
SRR7039197	6	7	10	8	9	12	11	294	*C. belfantii*

ST, sequence type
†When applicable

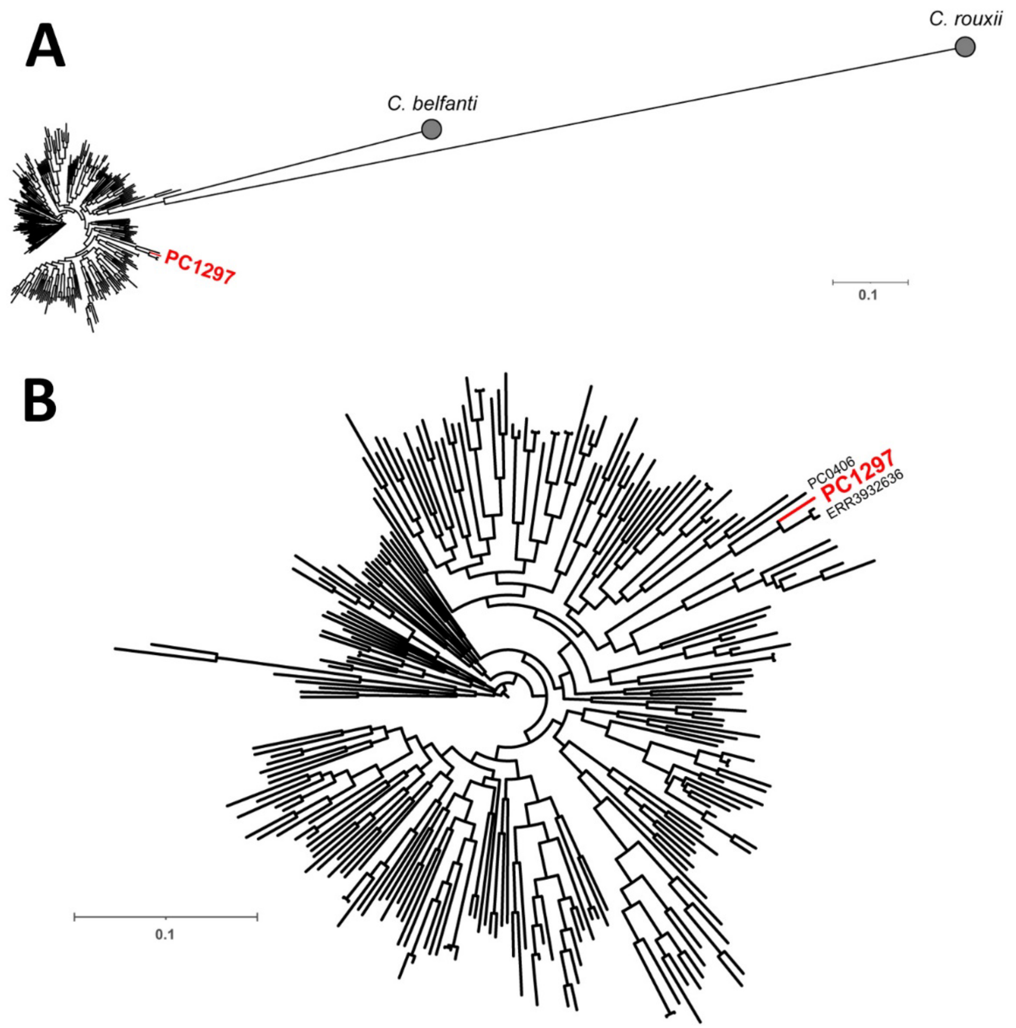

Appendix Figure. Phylogenetic reconstruction of *C. diphtheriae* PC1297 and 273 publicly available isolate sequences from 252,766 core SNP sites using maximum likelihood. Cat isolate PC1297 A) was placed within the main clade of typical *C. diphtheriae* strains, distinct from related novel species *C. belfanti* and *C. rouxii* previously associated with domestic dog and cat isolates, and B) was most closely related to human isolate ERR3932636 and shared a branch with dog isolate PC0406. Scale bars indicates substitutions per site.
